# Stress Impact of the Annealing Procedure of Cu-Filled TSV Packaging on the Performance of Nano-Scaled MOSFETs Evaluated by an Analytical Solution and FEA-Based Submodeling Technique

**DOI:** 10.3390/ma14185226

**Published:** 2021-09-11

**Authors:** Pei-Chen Huang, Chang-Chun Lee

**Affiliations:** Department of Power Mechanical Engineering, National Tsing Hua University, No. 101, Section 2, Kuang-Fu Road, Hsinchu 30013, Taiwan; mars420225@gmail.com

**Keywords:** MOSFET, TSV, annealing process, finite element analysis, carrier mobility estimation

## Abstract

Stress-induced performance change in electron packaging architecture is a major concern when the keep-out zone (KOZ) and corresponding integration density of interconnect systems and transistor devices are considered. In this study, a finite element analysis (FEA)-based submodeling approach is demonstrated to analyze the stress-affected zone of through-silicon via (TSV) and its influences on a planar metal oxide semiconductor field transistor (MOSFET) device. The feasibility of the widely adopted analytical solution for TSV stress-affected zone estimation, Lamé radial stress solution, is investigated and compared with the FEA-based submodeling approach. Analytic results reveal that the Lamé stress solution overestimates the TSV-induced stress in the concerned device by over 50%, and the difference in the estimated results of device performance between Lamé stress solution and FEA simulation can reach 22%. Moreover, a silicon–germanium-based lattice mismatch stressor is designed in a silicon p-type MOSFET, and its effects are analyzed and compared with those of TSV residual stress. The S/D stressor dominates the stress status of the device channel. The demonstrated FEA-based submodeling approach is effective in analyzing the stress impact from packaging and device-level components and estimating the KOZ issue in advanced electronic packaging.

## 1. Introduction

Moore’s law has been adopted for half a century, and it is still regarded as the target of transistor device performance. Silicon (Si) is the mainstream material for current semiconductor technology because of its low cost, mature fabrication process and acceptable performance. In the past decade, several advanced materials have been studied and used to replace Si as the new mainstream material in the semiconductor industry. Among the promising materials, germanium (Ge) and groups III–V are the most feasible due to their superior initial carrier transmission capability [1,2]. Strain engineering has been proposed to further enhance device performance under the same technology node through the lattice-mismatch mechanism. The four-point-bending technique is commonly utilized to extract the piezoresistance behaviors of device materials and estimate the stress-induced performance variation quantitatively [3,4,5]. Notably, the measured piezoresistance on bulk wafer and wafer with actual devices can differ considerably depending on the device type [3,5]. The stress sensitivity of different semiconductor materials has also been studied [4]. In electronic packaging architecture, the interconnect system plays an important role in signal transmission and delay time; notably, the overall delay time of an electronic packaging is determined by device and interconnect scaling [6]. Hence, the stability of the interconnect system is also an issue of electronic packaging. Through-silicon via (TSV) is the main interconnect architecture in 3D integrated circuit packaging, and the current mainstream TSV is fabricated with electroplated copper (Cu) [6,7,8,9,10,11]. Protrusion and thermal stress are the major mechanical reliability issues in TSV. The protrusion and thermal stress of TSV generally depend on the fabrication and annealing procedure and can generate cohesive and interfacial cracking on TSV [12,13,14,15,16]. Raman spectroscopy is widely used to estimate experimentally the residual stress of Cu TSV and the stress impact on the surrounding wafer [17,18,19]. In the fabrication procedure of Cu TSV, the annealing process is a critical step to manage the material characteristics, residual stress and Cu pumping. Cu annealing promotes interdiffusion, grain growth and re-crystallization to accomplish the abovementioned goals in thermomechanical reliability management. The TSV procedure is of three types, namely, TSV-first, TSV-middle and TSV-last, and the fabrication step of the via depends on the front-end (FE) and back-end (BE) processes. In the TSV-first procedure, the TSV is etched and filled, followed by FE and BE processes and wafer thinning. In comparison, TSV etching and filling are performed between the FE and BE in the TSV-middle procedure, and the TSV-last procedure means the FE/BE processes and wafer thinning are performed before TSV fabrication. The annealing effects on Cu’s characteristics, including microstructure, elastic modulus and hardness, have been explored [20]. The critical temperature of zero stress impact transferred from Cu TSV to the surrounding Si wafer has been studied through simulation and experimental measurement [21,22,23]. For the diameter-dependent stress status of narrow Cu TSV, whose diameter is below 8 μm, the measured mean hydrostatic stress ranges from 150MPa to 200 MPa [24]. In a previous study, residual tensile stress values of 234 and 167 MPa before and after 200 °C of annealing, respectively, were separately extracted through synchrotron X-ray microdiffraction, and the mechanism of residual stress relaxation was generated by the lattice reorganization behavior [25]. Another study revealed that residual stress can be increased to around 600–700 MPa after 420 °C of annealing and cooling down to room temperature [26]. A transient selective annealing technology was presented in another work, and its influence on the thermomechanical reliability of Cu TSV was analyzed [27]. The layout design dependence of the thermomechanical behavior of TSV has also been investigated [28,29,30]. The concept of keep out zone (KOZ) has been proposed to obstruct the stress influence of TSV on the surrounding wafer and improve the transistor performance, and many structural and material designs have been studied since then. A novel structural design of TSV called annular-trench-isolated TSV was designed in previous research to reduce the volume of filled Cu and decrease the corresponding coefficient of thermal expansion (CTE, *α*) mismatch between Cu TSV (*α* =16.7 ppm/K) and Si (*α* =2.3 ppm/K) wafer [31,32]. In the TSV architecture, the barrier is adopted to prevent Cu diffusion into the surrounding wafer, and several barriers can function as stress buffer layers at the same time [33,34,35,36,37,38,39]. Different barrier materials with various deposition pressures and rates have been investigated [38]. The advantages of using metal-based barriers in Cu TSV protrusion and thermal stress have been studied [39]. The results have shown that a barrier with high modulus and similar CTE as that of Cu can significantly reduce the protrusion of TSV, but high stresses transfer from TSV to the surrounding Si. Several analytical formulas have been utilized to investigate the stress impact of the TSV core and adjacent region, and their results have been compared with experimental and simulation results [40,41,42,43]. However, previous studies on KOZ estimation generally considered the actual transistor as a simple bulk Si. This means the layout design of the nano-scaled device was not considered, and the stress transfer efficiency from the TSV core to the device might have been overestimated or underestimated. A finite element analysis (FEA)-based submodeling technique is developed in this study, and its results are compared with analytical results. Moreover, device strain engineering is considered in the constructed FEA model for investigating the comprehensive performance change of the device under lattice strain and TSV residual stress.

## 2. Fundamental Theories of 2D Analytical Stress Solution, Lattice Stress Estimation Approach and Piezoresistance Behavior for Stress-Induced Performance Investigation

### 2.1. 2D Analytical Solution for Stress Estimation of TSV and the Surrounding Substrate

**Figure 1:**
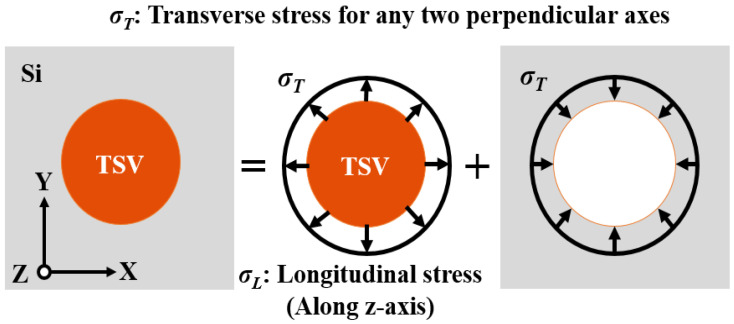
Schematic of Lamé radial stress solution based on the superposition principle. The stress field is integrated by the intrinsic stressed TSV core and reacted stress from the surrounding Si substrate.

**Figure 2:**
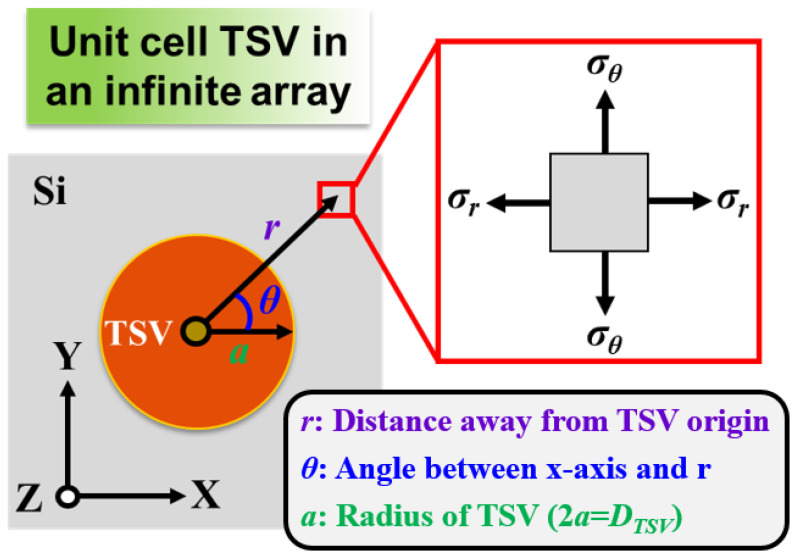
Schematic of TSV stress-affected zone calculation and corresponding structural parameters. The enlarged figure on the right side refers to the stress element in any location on the Si top surface.

The Lamé analytical stress model is widely adopted to estimate the influence of TSV-induced stress and the corresponding KOZ. The model assumes an infinite TSV surrounded by an infinite interposer when investigating the stress magnitude in the interposer under the impact of temperature change in the entire TSV interposer. A schematic of the Lamé radial stress solution is illustrated in Figure 1. Under the plane strain assumption, the entire structure is integrated with the core and surrounding material on the basis of the superposition principle. Assuming that the core is in a triaxial and uniform stress field, this stress field can be further separated into two stress components, namely, longitudinal stress (*σ_L_*) along the out-of-plane axis and transverse stress (*σ_T_*) for any two perpendicular axes. Through a derivation based on elasticity theory, the Lamé radial stress solution for the TSV stress-affected zone can be expressed as follows [40]:

(1) $$\sigma_{r}=\frac{-E_{Cu}(\alpha_{Cu}-\alpha_{Si})\Delta T}{1-2\nu_{Cu}+\frac{1+\nu_{Si}}{1+\nu_{Cu}}\frac{E_{Cu}}{E_{Si}}}{\left(\frac{D_{TSV}}{2r}\right)}^{2},$$

(2) $$\sigma_{r}=\sigma_{xx},\sigma_{\theta }=\sigma_{yy},\sigma_{r}=-\sigma_{\theta },$$

where *σ_r_* and *σ_θ_* denote radial and circumferential stresses, respectively. Figure 2 shows a detailed diagram of how TSV-induced stress influences the concerned device location. Labels *D_TSV_* and *r*, respectively, denote the diameter of TSV and the distance between the TSV origin and the concerned device location for KOZ estimation. *E*, *α* and *ν* pertain to Young’s modulus, CTE and Poisson’s ratio, respectively. ΔT is the temperature variation resulting from the fabrication and treatment process, and the ΔT considered in this study is generated from the annealing procedure.

These theoretical stresses are calculated based on analytical formulas integrated with structural and material parameters. On the basis of the foregoing equations, a semi-empirical formula with a similar form is presented for TSV stress-affected zone estimation. The semi-empirical formula is written as follows [19]:

(3) $$\sigma_{Si,xx}=\sigma_{TSV}\cdot (\cos^{2}\theta -\sin^{2}\theta )\cdot {\left(\frac{D_{TSV}}{2r}\right)}^{2},$$

(4) $$\sigma_{Si,yy}=\sigma_{TSV}\cdot (\sin^{2}\theta -\cos^{2}\theta )\cdot {\left(\frac{D_{TSV}}{2r}\right)}^{2}.$$

The advantage of this form is that the experimentally measured residual stress of TSV can be interpolated directly. The sign *θ* is the angle between the x-axis and *r*.

Many assumptions are made in the aforementioned model and cause a significant difference from the real TSV interposer architecture. First, the analytical model only considers the filler material of TSV and surrounding interposer and does not include the components adjacent to the core of TSV. Second, the analytical model does not consider the relative orientation between the concerned device channel and TSV core. These simplifications influence the efficiency of stress transfer from the TSV core to the surrounding components from a mechanical perspective and make the accuracy of the estimated performance variation in excepted transistor location controversial. Accordingly, this research utilizes the FEA-based submodeling simulation approach to explore the stress impact of the TSV core on the surrounding interposer and compares this approach with the classic Lamé radial stress solution.

### 2.2. Theotical Calculation of Lattice Mismatch Strain on the Absis of Vegard’s Law

Lattice mismatch strain simulation is performed in the present study to estimate the efficiency of lattice strain in device-level design and further compare it with the TSV stress-induced KOZ effect from the packaging-level structure. Lattice strain generation concentrates one element into another one to introduce volume expansion/shrinkage and corresponding stress; this phenomenon is approximated as the thermal stress mechanism. Accordingly, the virtual thermal strain approach has been proposed and validated, and it can be utilized to simulate lattice mismatch strain [44]. Its accuracy and feasibility have been validated through a comparison with literature data and analytically derived stress/strain formulas [45,46]. The lattice constant of concerned materials is calculated to estimate the subsequent lattice mismatch strain. Consequently, the formula for lattice parameter estimation of the concentrated component is computed in accordance with Vegard’s law as follows:

(5) $$a_{A}{}_{{}_{1-x}B_{x}}=a_{A}\times (1-x)+a_{B}\times (x),$$

where a_A_ and a_B_ refer to the lattice constant of the materials in pure form and $a_{A}{}_{{}_{1-x}B_{x}}$ is the lattice constant of the combined components mixed by pure materials A and B. The sign x denotes the mole fraction of concentrated material B. Thus, the lattice mismatch strain amount, defined as parameter f, can be estimated as follows:

(6) $$f=\frac{a_{A}-a_{A}{}_{{}_{1-x}B_{x}}}{a_{A}{}_{{}_{1-x}B_{x}}}.$$

From the abovementioned equation, the lattice mismatch strain generated from the designed lattice mismatch stressor is estimated and can be used to investigate the influence on device performance through the utilization of piezoresistance behavior.

### 2.3. Piezoresistance Characteristics of Semiconductor Material for Estimating Stress-Induced Carrier Mobility Variation

Piezoresistivity is a material characteristic in terms of stress and electric resistance. From the electric performance view, the increment in performance is proportional to the decrease in electric resistance. Accordingly, the approximation effect of stresses on device performance can be estimated through the integration of stresses in the device channel region and piezoresistance parameters. The detailed formula is expressed as follows:

(7) $$\frac{\Delta \mu }{\mu_{0}}=-\frac{\Delta \rho }{\rho_{0}}=-(\sigma_{xx}\pi_{xx}+\sigma_{yy}\pi_{yy}),$$

where Δ*μ* and *μ*_0_, respectively, denote the stressed and initial mobilities of the device channel. Parameters *π* and *σ* are the piezoresistivity coefficient along the concerned direction of the device channel. With reference to the piezoresistance of the Si transistor, the piezoresistance values in longitudinal (*x*-direction) and transverse (*y*-direction) directions are significantly larger than that in the vertical (*z*-direction) direction. Two major stress components (*σ_xx_* and *σ_yy_*) of the device channel are extracted to investigate the stress-induced carrier mobility variation in Si pMOSFET in this study. The piezoresistance coefficients of Si pMOSFET for mobility change calculation are obtained from Literature [5].

## 3. FEA Modeling of Global TSV Interposer Packaging Architecture and Local Transistor De-Vice Model Based on the Submodeling Technique

**Figure 3:**
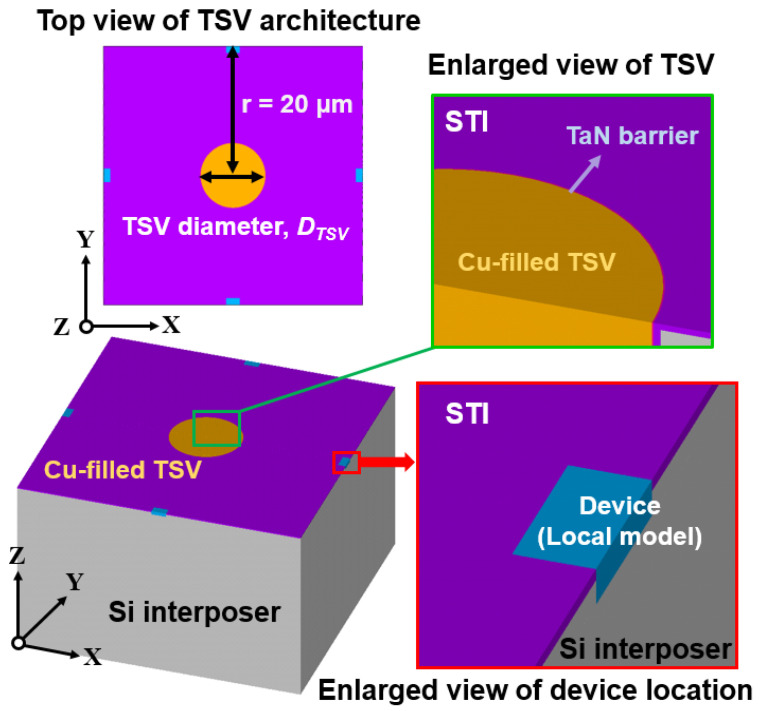
Schematic of a single TSV unit FEA model in an array-type TSV interposer and the corresponding structural parameters and materials. The TSV pitch is designed as 40 μm in this study.

Generally, the submodeling technique is a modeling approach to overcome the difficulty in the modeling and meshing of an FEA model with a significant size difference between concerned components. In this study, the approach is utilized to consider the stress influence and transfer efficiency of a micro-scaled TSV interposer and a nano-scaled transistor device in the same model. The micro-scaled TSV interposer is defined as the global model in the present submodeling procedure and illustrated in Figure 3. A single TSV unit in the entire symmetric array-type TSV interposer is extracted and constructed as an FEA model. The TSV unit model is composed of Cu-filled TSV, adjacent titanium nitride barrier layer and surrounding silicon dioxide (SiO_2_) dielectric layer as shallow trench isolation (STI). The designed thickness of STI and the barrier layer are 0.16 μm and 40 nm, respectively. Notably, the features of STI prevent electronic signal leakage, and the barrier layer prevents the diffusion of the TSV core material from contaminating the surrounding Si interposer. In this study, the distance between the origin of the TSV core and the concerned device location is fixed at 20 μm (labeled as symbol r in Figure 3); this value is also referred to as the half of TSV pitch, and the TSV depth is fixed at 50 μm. Several TSV diameters, namely, 5, 10, 15 and 30 μm, are designed to estimate the stress influences on the transistor device by using the analytical Lamé radial stress solution and FEA-based submodeling simulation approach.

**Figure 4:**
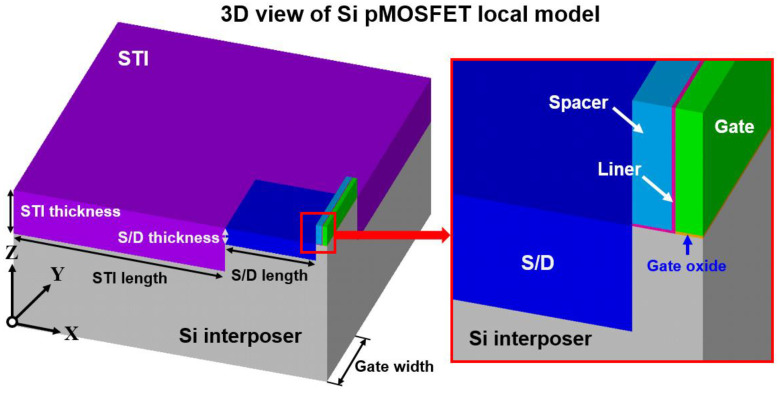
Schematic of a local Si pMOSFET FEA model and the corresponding structural parameters and components.

In the global TSV interposer model, the characteristics of the device region (labeled as the local model in Figure 3) is considered similar to the material of the device channel, Si, in this study. According to the procedure of the submodeling technique, a detailed transistor device local model also needs to be constructed, and the corresponding transistor model is shown in Figure 4. A half-symmetry FEA model of the device is constructed, but only a quarter model is illustrated in Figure 4 to introduce the structural parameters and structure components clearly. The components of the gate stacking structure of the device include the gate, liner, spacer and gate oxide, and the corresponding materials of the components considered in this study are poly-Si, SiO_2_, silicon nitride and SiO_2_, respectively. The thickness of the spacer, liner and gate oxide are 20, 2 and 1.5 nm, respectively. The heights of the gate and spacer are 70 nm, and the length of the gate is in accordance with the technology node of the 28 nm transistor device. The concerned device is fabricated on the (001)[110] lattice orientation of Si wafer. 

**Figure 5:**
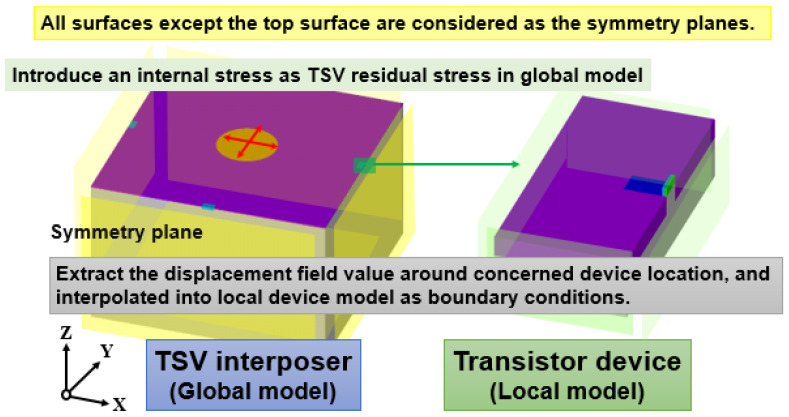
Schematic of how to introduce the TSV stress influence into the transistor device region based on the submodeling technique.

A general design for lattice strain generation incorporates the favorable element into the base material. In the Si pMOSFET architecture, Ge is adopted in Si and forms the silicon–germanium (Si_1−x_Ge_x_) concentrated alloy in the source/drain (S/D) region, which is a widely used and effective approach to introduce the preferred stress status of Si pMOSFET. The concentration of Ge in the Si_1−x_Ge_x_ stressor in this study is fixed at 25%, which is the most common design for Si pMOSFET. The S/D’s length and thickness are fixed at 300 and 60 nm, respectively. As the functional isolation, STI length and thickness are fixed at 700 and 160 nm, respectively. Hence, the constructed local model can be regarded as a single unit in a device array layout. The gate width of the device channel is the major design parameter in analyzing the gate width dependence on stress influence from TSV residual stress and the relationship of lattice mismatch stress with performance variation. After constructing the global TSV interposer model and local transistor device model, the procedure of submodeling for linking the mechanical response between the TSV interposer and transistor device is illustrated in Figure 5 and described in detail as follows. All surfaces, expect for the top surface of the TSV interposer model, is regarded as symmetric planes for boundary condition consideration. 

The annealing process-induced stress field of the TSV interposer should be generated in the FEA model by incorporating the considered residual stress (RS) magnitudes, 167 and 700 MPa, which, respectively, refer to the RS of Cu-filled TSV after 200 °C [25] and 420 °C [26] of annealing. Then, the displacement field around the device region of the global TSV interposer model is extracted and further interpolated into the local device model. Afterward, the TSV’s RS impact is introduced to the concerned Si pMOSFET and can be integrated with the S/D lattice strain stressor comprehensively. The mechanical characteristics of all materials and the parameters for annealing stress calculation for analytical solution and FEA simulation are summarized in Table 1.

## 4. Results and Discussions

### 4.1. Comparison of Analytical and FEA Submodeling Results on the Longitudinal and Trasnverse Stress of the Si pMOSFET Device Channel

**Figure 6:**
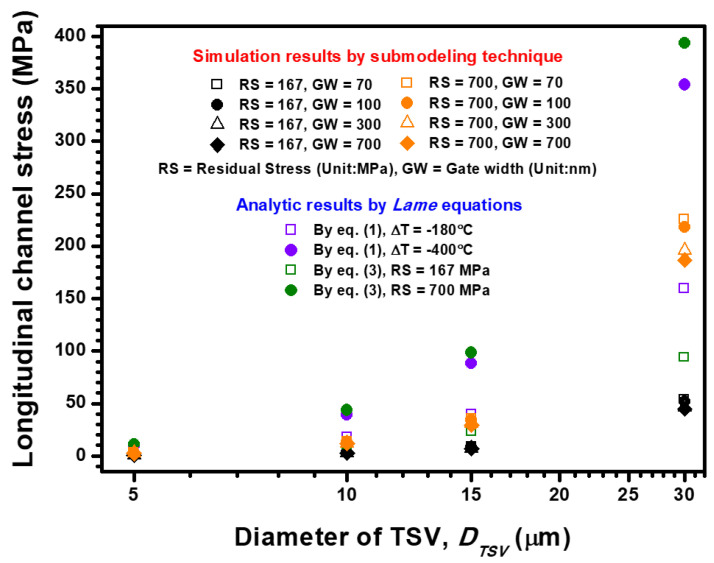
TSV-diameter, TSV-residual stress magnitude and channel gate width dependence longitudinal channel stress estimation (stress along the channel length direction) by using the analytical formula, semi-empirical formula and FEA submodeling simulation.

The feasibility of the analytical/semi-empirical stress estimation formula and its difference from the FEA submodeling results are explored. From the viewpoint of the piezoresistance of Si pMOSFET, the stress sensitivity in the vertical direction of the device channel is at least 13 times lower than those in the longitudinal and transverse directions [10]. Moreover, because the 2D Lamé stress model cannot generate the analytic results of vertical directional stress, the two other major stress components (longitudinal and transverse stresses) are estimated and discussed using the aforementioned approaches. The TSV diameter-dependent channel stress is calculated and illustrated in Figure 6. Considering the 420 °C annealing process’ impact on Cu-filled TSV with 30 μm diameter, the stress-free temperature is defined as the designed annealing temperature and subsequently cooled down to room temperature of 200 °C. The parameters ΔT = −400 °°C and RS = 700 MPa are, respectively, interpolated into Equations (1) and (3). Thus, the estimated longitudinal stress introduced into the device channel provided by Equations (1) and (3) is 353.95 and 393.75 MPa, respectively. The two analytic results show similar magnitudes under the same temperature loading condition. The calculated stress magnitudes reveal that the stress estimation feasibility of Equations (1) and (3) is highly comparable, and a 10% difference exists between the pure analytic result [from Equation (1)] and semi-empirical formula result [from Equation (3)]. Moreover, the calculated results from Equation (1) are lower than the results from Equation (3) for all designed TSV diameters, but the variation for the narrow TSV whose diameter is less than 15 μm is small. 

When the 200 °C annealing process-induced stress impact (ΔT = −180 °C and RS = 167 MPa) generated by the 30 μm Cu-TSV on the Si device is considered, the estimated longitudinal stress in the device channel based on Equations (1) and (3) is calculated as 159.28 and 93.94 MPa, respectively. An opposite trend is observed compared with the situation that considers the 420 °C annealing stress impact. This phenomenon can be attributed to the pure analytic formula, which relies on the assumption that Cu-TSV is ideally stress-free under the considered annealing temperature. However, extant literature indicates that RS is not effectively relaxed by only the 200 °C annealing procedure, and −196 MPa (the minus mark refers to the compressed stress status) is measured at 200 °C [25]. Meanwhile, almost zero stress at 420 °C was experimentally obtained in another study [26]. Accordingly, the pure analytic and semi-empirical formulas show reasonable consistency when a high annealing temperature of over 400 °C is considered. However, when a relatively low-temperature (below 400 °C) annealing process is designed, the semi-empirical formula is more suitable for estimating the impact of RS on transistor device performance compared with the pure analytic formula. 

The FEA submodeling results are also presented in Figure 6 to compare the estimated stress magnitudes with the analytic stress results. In consideration of packaging-level and device-level layout design, TSV diameter and channel gate width, the FEA submodeling stress results are much lower than the stress magnitudes calculated from Equations (1) and (3). When the 30 μm TSV diameter and 70 nm gate width are designed, 225.77 MPa of longitudinal stress is introduced into the Si pMOSFET channel. This result means that the analytical and semi-empirical formulas overestimated 56% of the longitudinal channel stress compared with the FEA submodeling simulation result. This mechanism can be explained by the stress buffer behavior of the barrier and STI structure between the TSV core and Si pMOSFET. Moreover, the layout design of Si pMOSFET plays a role in TSV stress transfer efficiency. When the channel gate width increases from 70 nm to 700 nm, the introduced longitudinal channel stresses decrease from 225.77 MPa to 186.83 MPa. These results indicate that the increased gate width leads to an increment in the structural stiffness of Si pMOSFET and further obstructs the RS influence from the TSV introduced into the device channel. Thus, the effects of TSV diameter and device gate width on longitudinal channel stress are systemically discussed. 

**Figure 7:**
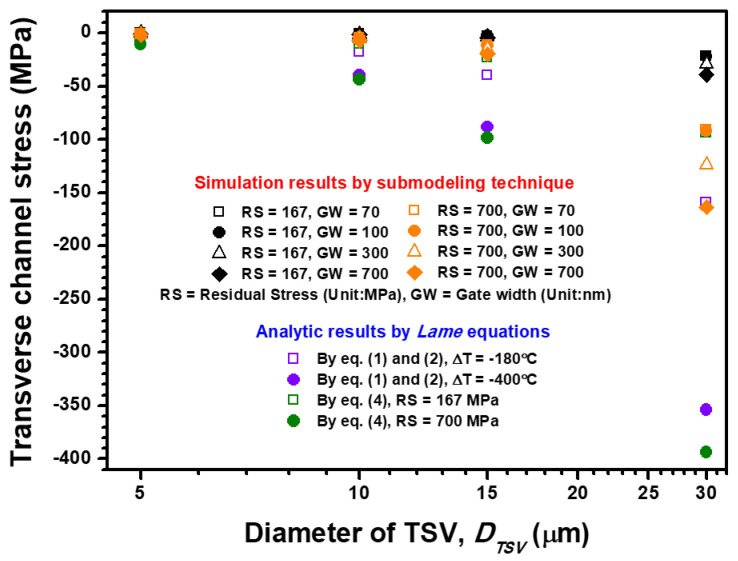
TSV-diameter, TSV-residual stress magnitude and channel gate width dependence transverse channel stress (stress along the channel width direction) estimated by the analytical formula, semi-empirical formula and FEA submodeling simulation.

The dependence of transverse channel stress on TSV diameter and channel gate width is illustrated in Figure 7. The same stress magnitude of transverse-direction channel stress but different stress status from tensile to compressive are calculated by the analytical and semi-empirical formulas. However, the channel gate width shows a positive influence on the increment in compressive transverse channel stress. The transverse channel stress is enhanced from −90.63 MPa to −163.52 MPa when the channel gate width increases from 70 nm to 700 nm. These results reveal that the enlarged gate width is beneficial to the stress obstruction in the longitudinal direction, but it aggravates the compression in the transverse direction because of its slim geometry with a large aspect ratio. The distance-to-radius ratio is also an important parameter to investigate annealing-induced thermal stress from TSV. In this study, the TSV pitch is fixed at 40 μm, which means the foregoing ratio is managed by the variation of TSV diameter. The distance-to-radius ratios of the four designed TSV diameters are calculated as 7, 3, 1.66 and 0.33, which correspond to 5, 10, 15 and 30 μm diameters, respectively. These ratios are attributed to the estimation of the critical criteria, which prevent the harsh thermal stress impact of TSV on the concerned device. For longitudinal and transverse channel stresses, the stress impact is suddenly increased when a distance-to-radius ratio of below 1 is considered. Accordingly, the aforementioned ratio is crucial for thermal stress management. On the basis of the piezoresistance of Si pMOSFET, the tensile and compressive stresses have a negative effect on stress-induced performance, which means the optimized gate width should be designed carefully to minimize the KOZ region under the annealing RS impact of Cu-filled TSV. Thus, KOZ estimation of Si pMOSFET is presented and discussed in the following section.

### 4.2. Stress-Induced Hole Carrier Mobility Gain Change and KOZ Estimation Based on Analytical and FEA Submodeling Results

**Figure 8:**
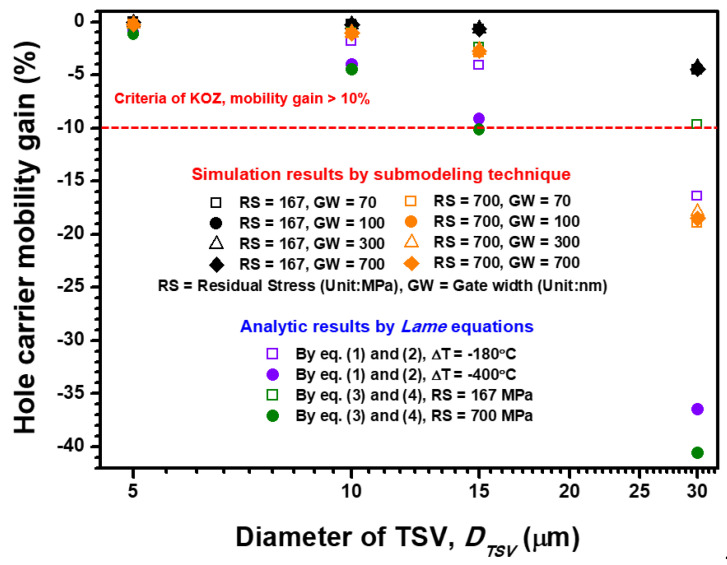
Comprehensive stress-induced impact on Si pMOSFET performance under different combinations of TSV/device layout design and TSV residual stress magnitude. The hole carrier mobility gain is estimated by the simulated stress components integrated with the piezoresistive behavior of Si.

When the distance between the TSV origin and Si pMOSFET is fixed at 20 μm, the criterion for KOZ determination is considered to be a 10% change in carrier mobility gain [45]. As shown in Figure 8, the hole carrier mobility gain change is apparently unfavorable for the 30 μm TSV diameter design with 700 MPa RS. Carrier mobility gains of −36.46% and −40.56% are estimated by the analytical solution and semi-empirical formula, respectively. In comparison, the FEA submodeling results reveal nearly −18% hole carrier mobility gain change under the same TSV diameter and RS magnitude. This nearly −18% mobility gain change is not linearly proportional to the designed gate width because the longitudinal and transverse stress-induced mobility gain changes compete with each other. For the TSV diameter below 15 μm, the estimated mobility gain changes meet the design criteria of KOZ determination (10% mobility change) under 700 MPa RS impact (corresponding to the 420 °C annealing procedure). Notably, the nearly 22 μm TSV diameter design is acceptable according to the FEA submodeling results. The KOZ region can be further determined by the distance between the designed *r* (20 μm) and the edge of TSV. Therefore, the KOZ regions are, respectively, estimated as 12.5 μm (for *D_TSV_* = 15 μm) and 9 μm (for *D_TSV_* = 22 μm) by the analytical solution/semi-empirical formula and FEA submodeling approach. Moreover, the estimated results provided by the semi-empirical formula are similar to the FEA submodeling results when 167 MPa RS magnitude and 15 μm TSV diameter are considered; however, a −1.8% mobility change is still overestimated. Moreover, the mobility gain variation is almost independent of the designed *D_TSV_* when the 167 MPa RS is considered. This phenomenon can be attributed to the piezoresistive behavior of Si pMOSFET, and the compressive and tensile stresses are attributed to the stress-induced performance. However, the longitudinal and transverse channel stresses are positively and negatively proportional to the increment of *D_TSV_*, respectively, which means the positive and negative influences on device performance generated by longitudinal and transverse stresses are countervailed. Accordingly, the final carrier mobility gain is almost independent of channel gate width variation. These results indicate that the estimated hole carrier mobility change provided by the analytical solution and semi-empirical formula is significantly overestimated compared with the value from the FEA submodeling approach, which considers the actual structural characteristics of the layout design on packaging and device levels. These results are beneficial to accurately estimating the KOZ region and further increasing the integration density from packaging-level design in the TSV interposer architecture.

### 4.3. FEA Submodeling Results on the Longitudinal and Transverse Stresses of the Si pMOSFET Device Channel under the Integrated Effect of TSV RS and Strain Engineering S/D Lattice Stressor

**Figure 9:**
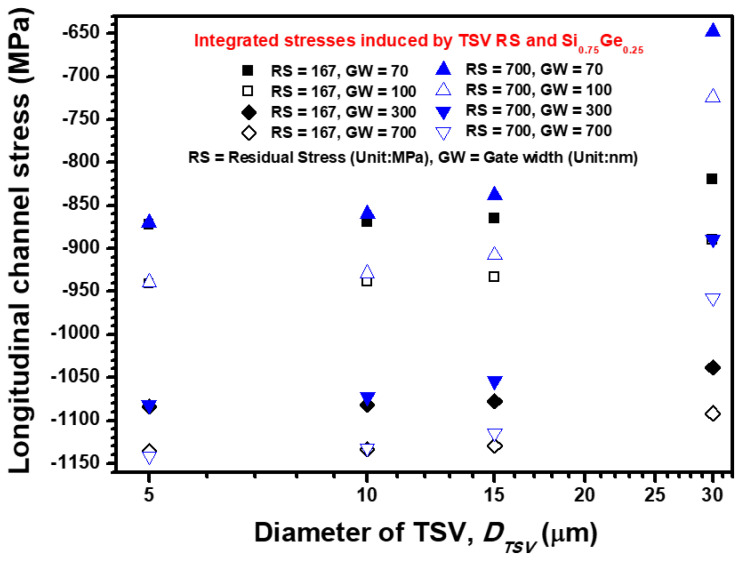
Dependence of the layout and annealing process design on the longitudinal channel stress of Si pMOSFET estimated by the analytical formula, semi-empirical formula and FEA submodeling simulation approach.

The influence of TSV RS on device performance is systemically discussed in this section. On the basis of an unstrained Si pMOSFET, the TSV RS-induced performance degradation is estimated to be 0.05 % to 18.93 % depending on the designed TSV diameter and channel gate width. This phenomenon is not favorable because the performance degradation of pMOSFET is difficult to determine using nMOSFET with a high initial carrier transport capability. For this reason, the S/D lattice-strained Si_0.75_Ge_0.25_ stressor is used in this study to analyze the comprehensive effect on stress-induced performance variation under the integrated stresses of TSV RS and S/D lattice mismatch. As shown in Figure 9, the S/D lattice stress dominates the longitudinal stress magnitudes in the Si pMOSFET channel. This dominance is attributed to the high stress transfer efficiency between S/D and the device channel in accordance with the direct contact between the foregoing components. According to the analytic results presented in Figure 7, the longitudinal channel stress impact introduced by 5 μm diameter TSV is almost zero. Hence, the longitudinal stress magnitude for the S/D strained Si pMOSFET with 5 μm TSV diameter (shown in Figure 9) can be regarded as the lattice mismatch stress generated by the S/D stressor. The lattice mismatch strain amount is proportional to the increment in gate width because an increased gate width enlarges the width of the S/D region and generates more lattice mismatch strain subsequently. For a narrow TSV design with a 5 μm diameter, the longitudinal channel stress is completely determined by the S/D lattice stress when the two different TSV RS magnitude (167 and 700 MPa) are considered. However, given that the design TSV diameter is enlarged from 5 μm to 30 μm, the longitudinal channel stress varies from −870.43 MPa to −647.66 MPa for a Si pMOSFET with a 70 nm gate width. These results are due to the enlarged TSV diameter shrinking the relative distance between TSV and the device location, thereby enhancing the tensile RS impact of TSV introduced into the device channel and weakening the compressive stress induced by the S/D stressor. Moreover, the narrow gate width cannot obstruct the tensile RS impact of TSV transferred to the concerned Si pMOSFET channel. Accordingly, the longitudinal channel stresses of lattice-strained Si pMOSFET are mainly dominated by the S/D stressor, but the RS impact of TSV also plays an important role when enlarged TSV diameters and RS magnitudes are utilized. 

**Figure 10:**
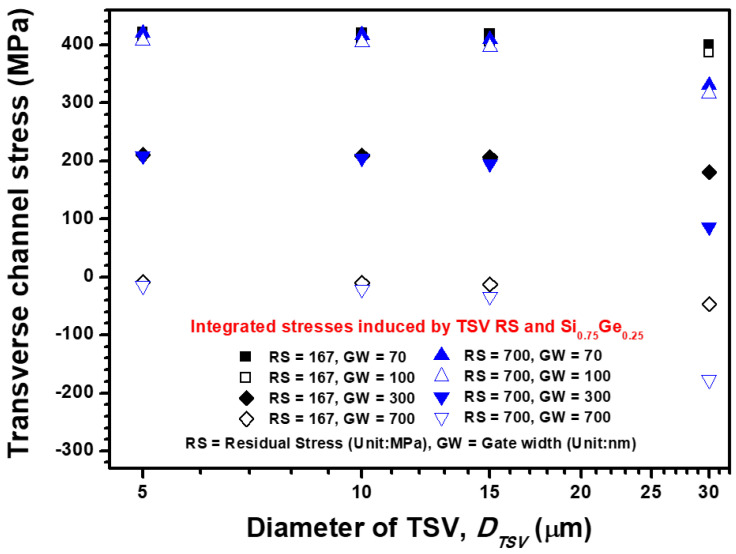
Dependence of the layout and annealing process design on the transverse channel stress of Si pMOSFET estimated by the analytical formula, semi-empirical formula and FEA submodeling approach.

The transverse channel stress introduced by the TSV RS impact and S/D strained Si_0.75_Ge_0.25_ stressor is further illustrated in Figure 10. Similar to the stress trend shown in Figure 9, the transverse channel stress remains stable because the RS impact generated by the Cu-filled TSV is limited to almost zero when the TSV diameter of below 15 μm is utilized. For a narrow gate width of 70 nm, a significant tensile transverse channel stress is observed. This stress status can be attributed to the Poisson’s ratio mechanism based on generalized Hooke’s law. When the gate width is enlarged from 70 nm to 700 nm, the aspect ratio of the device channel increases and limits the Poisson’s ratio mechanism. The enlarged gate width also extends the width of the channel and further degrades the uniformity and concentration of S/D-induced lattice mismatch stress. Notably, the compressive and tensile stress status along the longitudinal and transverse directions is favorable for stress-induced performance enhancement in accordance with the piezoresistance characteristics of Si pMOSFET. The optimized mobility gains of the considered Si pMOSFET are not linearly proportional to the increase or decrease in gate width. Thus, the gate width-dependent carrier mobility gains under the integrated stress generated by RS of TSV and the S/D lattice stressor are estimated and discussed in the following section.

### 4.4. Stress-Induced Hole Carrier Mobility Gain Change in the S/D Lattice-Strained Si pMOSFET Under the RS Impact Generated by Cu-Filled TSV 

**Figure 11:**
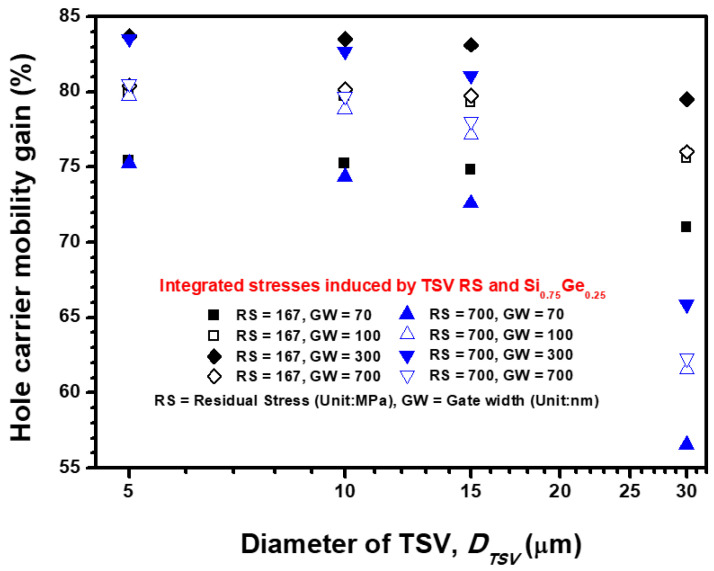
Comprehensive hole carrier mobility gain variation in the S/D lattice-strained Si pMOSFET.

On the basis of the longitudinal and transverse channel stresses presented in Figure 9 and Figure 10, the stress-induced carrier mobility gain generated by the RS of TSV and lattice mismatch stress of the S/D Si_0.75_Ge_0.25_ stressor is illustrated in Figure 11. The RS of TSV significantly degrades the hole carrier mobility of Si pMOSFET, and its influence is proportional to the increment in the designed TSV diameter and RS magnitude. In accordance with the gate width-dependent stresses discussed in the previous section, the optimized gate width is determined to be 300 nm. In consideration of the 420 °C annealing procedure for TSV (corresponding to 700 MPa RS) and 300 nm gate width for Si pMOSFET, the carrier mobility gain varies from 83.54% to 65.89% when the TSV diameter is enlarged from 5 μm to 30 μm. A −17.65% difference in carrier mobility gain results from the RS impact of TSV, and a similar change of nearly −18% to −19% is observed for all designed gate widths. Notably, the adopted piezoresistance parameter is regarded as constant, but in actual experimental measurements on piezoresistance extraction from transistors, it is simultaneously influenced by the doping density of the device channel, gate effective field, applied drain voltage and measurement uncertainty. On the basis of the piezoresistance of the Si pMOSFET in Literature [5], a maximum of 20% estimation uncertainty is explored. These results reveal that the performance of lattice-strained Si pMOSFET is dominated by the designed S/D stressor adjacent to the concerned device channel. However, a narrow TSV diameter and pitch design are the main factors for further increasing the integration density in electronic packaging. The TSV-induced stress impact on the performance of an advanced device will be harsh if the relative distance between TSV and the concerned device is narrowed to a few micrometers. The major contribution of the present study is that it demonstrates an FEA submodeling-based approach to estimate the stress-induced performance impact under the integration of packaging-level and transistor-level stresses. Moreover, the feasibility of the widely adopted analytical solution for TSV stress-affected zone estimation, the Lamé radial stress solution, and its derived semi-empirical formula are utilized and discussed in comparison with the present FEA submodeling approach. The analytic results presented in this study reveal that the abovementioned analytical solutions significantly overestimate the stress transfer efficiency from TSV to the device. The FEA submodeling technique demonstrated in this study provides an effective approach to analyze the stress-induced performance impact for high-integration-density design in electronic packaging and overcomes the difficulty of FEA model construction with a significant size difference between considered components from micro- to nano-level dimensions.

## 5. Conclusions

An FEA-based submodeling approach was demonstrated to estimate the stress impact from a packaging-level interconnect to a nano-scaled transistor device. The widely utilized Lamé radial stress solution and its derived semi-empirical formula were adopted to analyze the stress-affected zone generated by Cu-filled TSV design, and their results were compared with the results of the FEA-based submodeling approach. The analytic results revealed that the Lamé analytical solution overestimated the stresses transferred from TSV to the concerned device by over 50%. This result means that the stress transfer efficiency between TSV and the transistor device was overestimated because the stress buffer mechanism from the barrier, STI and the layout of the device are neglected in the abovementioned analytical solution. Under the same layout and RS impact of the designed TSV interposer packaging, −36.46%, −40.56% and −18% carrier mobility gains were estimated by the analytical solution, semi-empirical formula and FEA-based submodeling approach, respectively. The highly accurate estimation of the TSV stress-affected zone and its impact on device performance by the presented submodeling approach is due to the analysis of the KOZ region and increased integration density of the device with good usage of the wafer area. Moreover, the comprehensive stress impact generated by the RS of TSV and device-level strain engineering was investigated. The S/D lattice-strained Si_0.75_Ge_0.25_ stressor was utilized to generate a favorable stress status in Si pMOSFET, and its effect was compared with the RS impact from the Cu-filled TSV. The results showed the S/D lattice stressor dominated the status of stress components in the device channel, but the stress impact generated by TSV could be harsh if the integration density of devices and TSV interconnects is further increased. These issues can be further investigated using advanced 3D device architectures and the present FEA-based submodeling approach.

## Figures and Tables

**Table 1:** Material characteristics utilized in this study for analytical stress calculation and FEA stress simulation.

Model	Components	*E*, GPa	*ν*	*α*, ppm/K
Global TSV interposer	TSV (Cu)	117	0.30	16.7
	Barrier (TaN)	186	0.342	6.48
	Interposer (Si)	169	0.26	2.3
	STI (SiO_2_)	71.7	0.16	0.51
Local transistor device	Gate (Poly-Si)	160	0.22	2.3
	Liner (SiO_2_)	71.7	0.16	0.51
	Spacer (SiN)	123.3	0.30	3.05
	S/D (Si_0.75_Ge_0.25_)	161	0.265	3.2
	Substrate (Si)	169	0.26	2.3
	STI (SiO_2_)	71.7	0.16	0.51

## Data Availability

Data sharing is not applicable to this article.
